# Supplementary material for: CrPS4 Nanoflakes as Stable Direct-Band-Gap 2D Materials for Ultrafast Pulse Laser Applications
Source: Nanomaterials (Basel). 2023 Mar 22;13(6):1128. doi: 10.3390/nano13061128 (PMC10052116; doi:10.3390/nano13061128)
Supplement: Supplementary file 1 [file nanomaterials-13-01128-s001.zip › nanomaterials-2270930-supplementary.pdf]

Supplementary Materials

$CrPS_4$ Nanoflakes as Stable Direct-Band-Gap 2D Materials for Ultrafast Pulse Laser Applications

Wenyao Zhang[1,†], Yu Zhang[1,†], Xudong Leng[2], Qun Jing[2], Qiao Wen[1,*]

1 Key Laboratory of Optoelectronic Devices and Systems of Ministry of Education and Guangdong Province, College of Physics and Optoelectronic Engineering, Shenzhen University, Shenzhen 518060, China

2 Xinjiang Key for Laboratory of Solid State Physics and Devices, Xinjiang University, 777 Huarui Street, Urumqi 830017, China

* Correspondence: wenqiao@szu.edu.cn

† These authors contributed equally to this work.

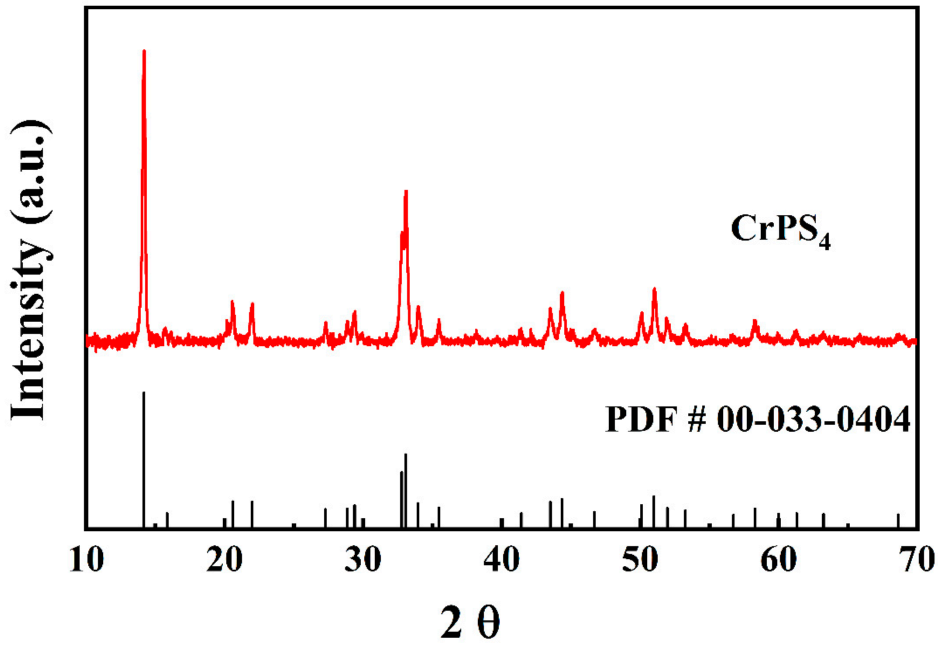

Figure S1. The diffraction pattern from $CrPS_4$ sample was analyzed using X′pert Highscore Plus 3.0

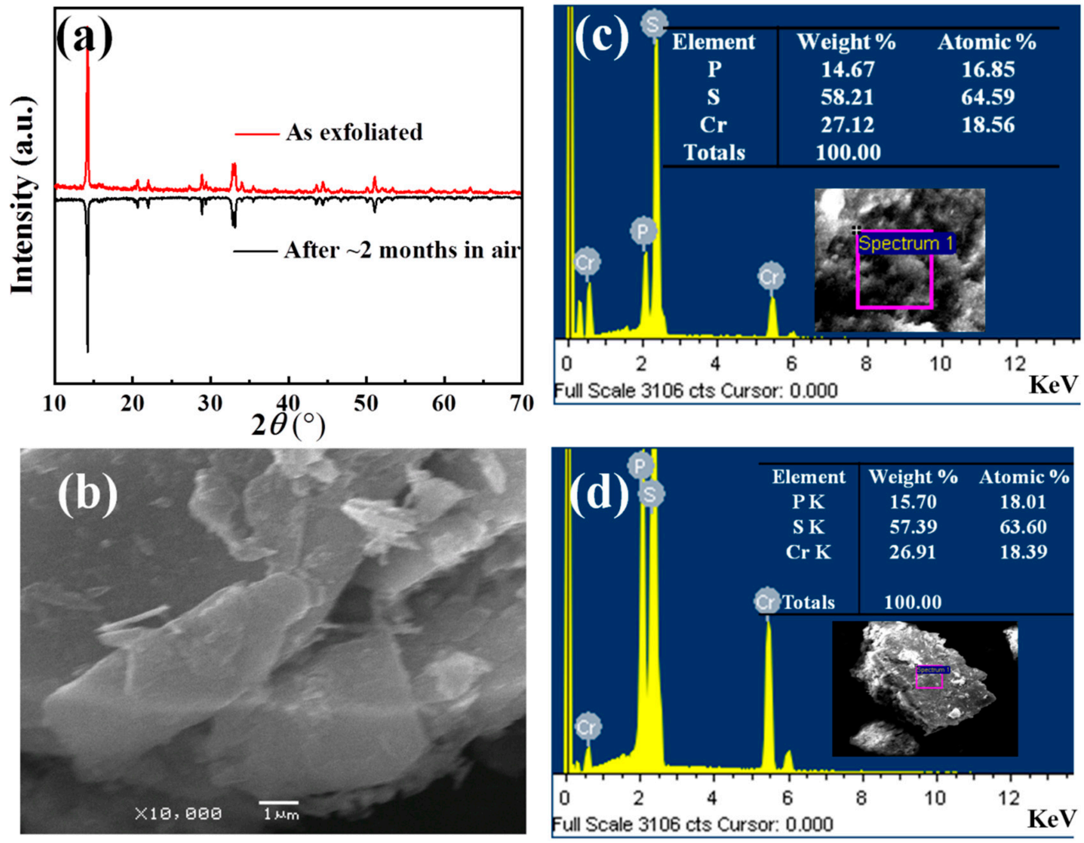

Figure S2. (a) XRD spectra of $CrPS_4$ as exfoliated and after ~2 months in air; (b) SEM image for $CrPS_4$ flakes after ~2 months in air; (d) EDX spectra with an atomic ratio of Cr, P and S elements for $CrPS_4$ flakes as exfoliated and after ~2 months in air.

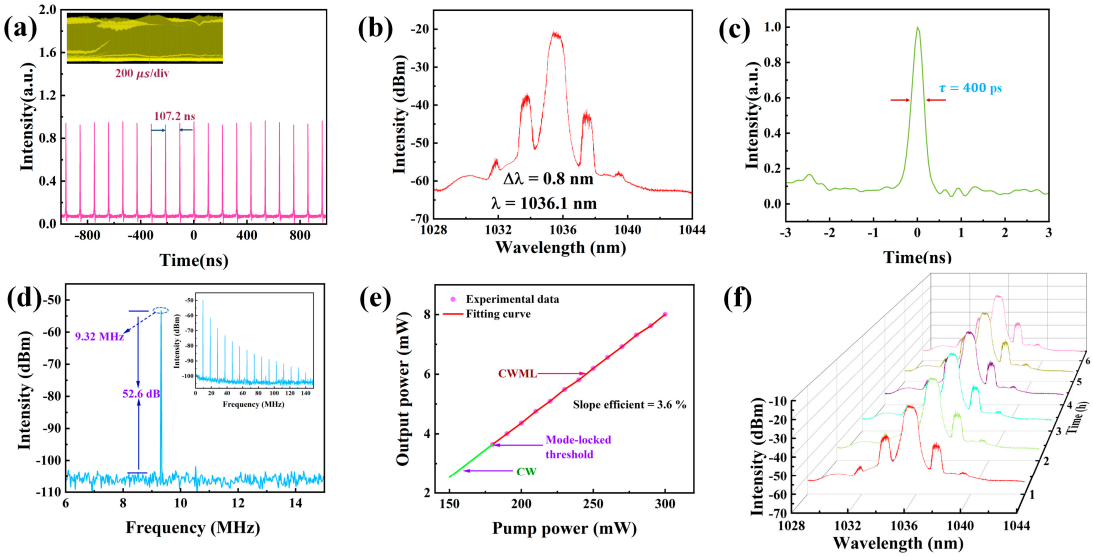

Figure S3. Classical mode-locked output characteristics. (**a**) Pulse train; (**b**) Optical Spectrum; (**c**) Pulse width; (**d**) RF spectrum (inset: broadband RF spectrum); (**e**) Relative change of output power and pump power; (**f**) Long-time spectroscopic measurement (1 hour interval, 6 hours in total).

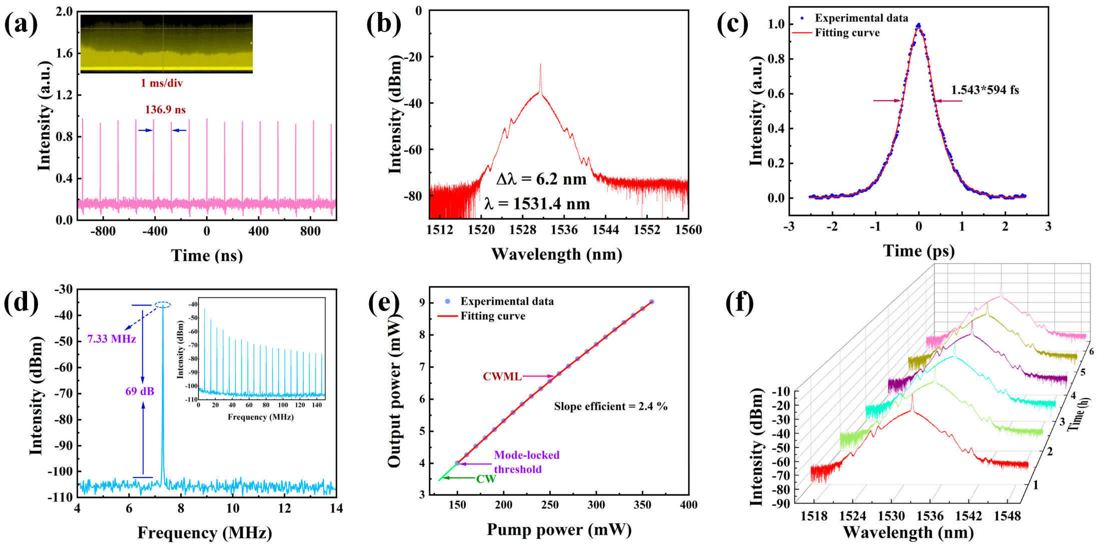

Figure S4. Classical mode-locked output characteristics. (**a**) Pulse train; (**b**) Optical Spectrum; (**c**) Pulse width; (**d**) RF spectrum (inset: broadband RF spectrum); (**e**) Relative change of output power and pump power; (**f**) Long-time spectroscopic measurement (1 hour interval, 6 hours in total).

Table S1. Comparison of SA with other 2D materials at 1 μm laser output parameters.

SA type	Pulse width (ps)	Output power(mW)	Pulse energy(nJ)	Peak power(W)	Ref.
**Graphene**	580	0.37	0.41	0.71	[54]
**Graphene oxide**	2300	0.19	0.18	0.08	[64]
**BP**	3.2	27	2.7	830	[65]
**BP**	400	32.5	0.7	1.75	[66]
**$Ti_3C_2T_x$**	480	9	0.47	1	[67]
**WS_2**	630	7.6	1.36	2.14	[56]
**Mo_2C**	350	7.7	0.38	1.09	[55]
**$MoSe_2$**	664.5	2	0.13	0.19	[68]
**MoS_2**	656	2.37	0.35	0.53	[69]
**MoS_2**	800	9.3	1.41	1.76	[70]
**Bi_2Te_3**	230	0.86	0.60	2.6	[53]
**$CrPS_4$**	298	10.63	1.174	3.94	**This work**

Table S2. Comparison of SA with other 2D materials at 1.5 μm laser output parameters.

SA type	Pulse width (fs)	Output power(mW)	Pulse energy(pJ)	Peak power(W)	Ref.
**Graphene**	756	2	1120	1478	[7]
**BP**	2180	0.077	4.9	2.3	[71]
**BP**	940	2.6	1129	1201	[57]
**Ti_3CNT_x**	159	3	410	2578.6	[67]
**Ti_3CNT_x**	660	0.05	3	4.9	[72]
**MoS_2**	200	1	68.8	2300	[73]
**MoS_2**	521	0.79	31.3	60.1	[74]
**WS_2**	21100	1.8	220	10.4	[56]
**Mo_2C**	290	6.831	860	2982	[55]
**In_2Se_3**	215	2	270	1255.8	[75]
**$CrPS_4$**	500	6.1	893	1786	**This work**
